# Impact of Hypertensive Heart Disease on Coronary Artery Disease Severity Assessed by the Duke Coronary Artery Disease Index

**DOI:** 10.7759/cureus.113892

**Published:** 2026-08-03

**Authors:** Fahrudin Sabanovic, Farisa Babic Sabanovic, Emir Becirovic, Amir Bećirović, Dragan Piljic

**Affiliations:** 1 Internal Medicine, Cantonal Hospital Zenica, Zenica, BIH; 2 Internal Medicine Clinic, Intensive Care Unit, University Clinical Center Tuzla, Tuzla, BIH; 3 Endocrinology, Internal Medicine, University Clinical Center Tuzla, Tuzla, BIH; 4 Cardiac Surgery, University Clinical Center Tuzla, Tuzla, BIH

**Keywords:** cardiac remodeling, coronary artery disease, duke cad index, hypertensive heart disease, revascularization, roc analysis

## Abstract

This retrospective study of 98 patients assessed whether hypertensive heart disease (HHD) is associated with angiographic coronary artery disease (CAD) burden, quantified by the Duke CAD Index, and whether HHD or CAD burden influenced revascularization strategy. HHD was independently associated with higher Duke CAD Index values in linear regression (B = 10.33, p = 0.038) and with greater odds of more severe CAD categories in ordinal regression (OR = 3.19, p = 0.015). However, revascularization choice did not differ by HHD status or CAD severity (p = 0.95). A model incorporating age, sex, diabetes mellitus, and HHD demonstrated moderate discriminative ability for predicting severe CAD (AUC = 0.753). These findings suggest that HHD identifies a high-risk coronary atherosclerotic phenotype, but they are hypothesis-generating and require confirmation in larger prospective studies before clinical application.

## Introduction

Hypertensive heart disease (HHD) represents a frequent manifestation of arterial hypertension and reflects cumulative pressure overload on the myocardium [1]. Prolonged exposure leads to left ventricular hypertrophy (LVH), concentric remodeling, impaired diastolic function, and left atrial enlargement [2]. Although initially compensatory, these changes increase myocardial oxygen demand and are thought to be associated with impaired coronary reserve and ischemic vulnerability [3].

Hypertensive cardiac remodeling frequently coexists with coronary artery disease (CAD) [4]. Sustained hypertension accelerates atherosclerosis, promotes endothelial dysfunction, and may influence the clinical course of CAD [5]. Prior studies have shown that LVH and hypertensive remodeling worsen coronary hemodynamics and are associated with poorer outcomes following ischemia or revascularization [6,7]. However, the relationship between the hypertensive remodeling phenotype and the angiographic extent of CAD remains insufficiently characterized. Furthermore, data regarding the influence of HHD on revascularization strategy selection are limited and inconsistent.

A clearer understanding of these relationships is clinically relevant, as both HHD and CAD contribute substantially to heart failure progression and cardiovascular morbidity [8,9]. Therefore, we aimed to compare angiographic CAD burden quantified by the Duke CAD Index between patients with and without HHD and to explore whether HHD status or Duke CAD Index categories are associated with revascularization strategy selection. Additionally, we sought to evaluate the discriminative ability of a clinical model including HHD for predicting severe CAD.

Although traditional cardiovascular risk factors such as hypertension, dyslipidemia, diabetes mellitus, obesity, and smoking remain central to risk stratification, there is growing recognition that social determinants of health, including socioeconomic status, race, education, income, employment, housing, food security, and psychosocial factors, act as independent and compounding risk factors for cardiovascular disease [10-12]. These factors not only influence the prevalence of traditional risk factors but also affect access to care, treatment adherence, and overall cardiovascular outcomes. Therefore, a comprehensive understanding of cardiovascular health requires consideration of both biological and social determinants.

We hypothesized that HHD is associated with a greater angiographic CAD burden and may be associated with revascularization strategy selection in this observational cohort. Patients without HHD served as the comparator group to provide a clinically relevant reference for evaluating the impact of HHD on CAD severity and treatment strategy.

## Materials and methods

Study design and population

We retrospectively screened 156 consecutive patients who underwent coronary angiography for stable suspected or established CAD at the Department of Cardiology, Cantonal Hospital Zenica, between January 2021 and December 2024. After applying the exclusion criteria, the final study cohort comprised 98 patients.

Inclusion and exclusion criteria

To be eligible for inclusion, patients were required to be over 18 years of age, hospitalized at the Department of Cardiology at Cantonal Hospital Zenica, and undergoing coronary angiography for suspected or established stable CAD. Complete clinical, echocardiographic, and angiographic documentation was also mandatory for eligibility. Patients were excluded if coronary angiography was indicated for acute coronary syndrome or if they had a history of prior coronary revascularization via percutaneous coronary intervention (PCI) or coronary artery bypass grafting (CABG). Additional exclusion criteria comprised significant valvular or congenital heart disease, moderate-to-severe aortic stenosis, hypertrophic cardiomyopathy, known infiltrative cardiomyopathies, uncontrolled arterial hypertension, incomplete echocardiographic records, and chronic kidney disease defined by an estimated glomerular filtration rate below 30 mL/min/1.73 m².

Of the 156 initially screened patients, 58 were excluded: 22 had incomplete echocardiographic data, 15 had prior revascularization, 12 had significant valvular disease, and 9 were excluded for other reasons (chronic kidney disease (n = 4), hypertrophic cardiomyopathy (n = 3), and uncontrolled hypertension (n = 2). The final cohort comprised 98 patients (45 with HHD, 53 without HHD).

Definition of HHD

We defined HHD according to the 2023 European Society of Hypertension guidelines and 2016 American Society of Echocardiography (ASE)/European Association of Cardiovascular Imaging (EACVI) recommendations as established arterial hypertension (blood pressure ≥140/90 mmHg or receiving antihypertensive therapy) combined with echocardiographic evidence of LVH [10,11]. We defined LVH by left ventricular mass index (LVMI) >115 g/m² for men and >95 g/m² for women.

The non-HHD group included both hypertensive patients without LVH (n = 30, 56.6%) and normotensive patients (n = 23, 43.4%). This approach was chosen because our primary objective was to identify the clinical phenotype of HHD (hypertension with LVH) as a distinct entity, rather than to isolate the effect of hypertension alone. Previous studies have demonstrated that LVH modifies cardiovascular risk independently of blood pressure level, suggesting that HHD represents a specific high-risk phenotype beyond hypertension itself [13,14]. Combining hypertensive patients without LVH with normotensive patients in the comparator group allows us to examine whether the additional burden of LVH identifies patients at higher risk, beyond the presence of hypertension alone. This approach is consistent with prior studies comparing HHD to non-HHD populations [3]. A sensitivity analysis comparing HHD patients to hypertensive patients without LVH only was performed and is reported in the Results.

Clinical and echocardiographic assessment

We obtained demographic data and cardiovascular risk factors from medical records. Dyslipidemia was defined as total cholesterol >5.2 mmol/L, LDL cholesterol >3.0 mmol/L, or current use of lipid-lowering therapy. Data on body mass index, serum creatinine, serum uric acid, and detailed medication use were not systematically recorded in this retrospective cohort; this represents a limitation.

Echocardiographic data extraction

Echocardiographic parameters were retrospectively extracted from the institutional electronic medical records. All echocardiographic examinations had been performed during routine clinical care by experienced cardiologists using a Philips EPIQ 7 ultrasound system (Philips Healthcare, Andover, MA, USA), following standard departmental protocols. Images had been acquired in the left lateral decubitus position using standard parasternal long-axis, parasternal short-axis, apical four-chamber, and apical two-chamber views. Measurements were averaged over three consecutive cardiac cycles in patients in sinus rhythm and over five cycles in patients with atrial fibrillation.

For this study, specific structural and functional parameters were systematically extracted from institutional electronic medical records. These parameters included left ventricular (LV) ejection fraction measured using Simpson's biplane method and left atrial diameter measured in the parasternal long-axis view. Additionally, LVMI was recorded, calculated using the Devereux formula and indexed to body surface area. Relative wall thickness (RWT) was also derived, calculated as twice the posterior wall thickness in diastole divided by the LV internal diameter in diastole.

LV geometry was classified into four categories according to the 2015 ASE/EACVI recommendations [14]: normal geometry (normal LVMI and RWT ≤0.42), concentric remodeling (normal LVMI and RWT >0.42), eccentric hypertrophy (increased LVMI and RWT ≤0.42), and concentric hypertrophy (increased LVMI and RWT >0.42).

Diastolic function parameters, left atrial volume index, and natriuretic peptide levels were not systematically available in the retrospective dataset and could not be included in the analysis.

Quality control for echocardiographic data

To ensure accuracy and reproducibility of the retrospectively extracted echocardiographic parameters, a quality control procedure was performed. Two independent investigators (D.P. and E.B.), both experienced cardiologists with more than 10 years of clinical practice, independently re-evaluated a random subset of 20 patients (approximately 20% of the cohort). The same echocardiographic parameters (left ventricular ejection fraction (LVEF), left atrial diameter, LVMI, and RWT) were re-measured from the stored digital images, with both investigators blinded to clinical and angiographic data.

For continuous variables, intra-observer and inter-observer variability were assessed using the intraclass correlation coefficient (ICC) and coefficient of variation (CV). The ICC values were 0.86 for LVMI, 0.82 for RWT, and 0.84 for left atrial diameter, indicating excellent agreement. The corresponding coefficients of variation for intra-observer variability were 3.8%, 4.1%, and 3.5%, respectively, and for inter-observer variability, 5.2%, 5.8%, and 4.7%, respectively. For the categorical classification of LV geometry, Cohen's kappa coefficient was 0.86, also indicating excellent agreement.

All disagreements were resolved by consensus or, when necessary, by discussion with a third experienced cardiologist. The original clinical reports were used for the primary analysis; the re-measurements served exclusively for quality assurance purposes and did not replace the original data.

Coronary angiography and Duke CAD Index

Coronary angiographic data were obtained from the hospital medical database. Coronary angiography had previously been performed using a standard femoral or radial approach with 6F or 7F catheters. All angiograms were acquired on a Philips Allura Xper FD10 system (Philips Healthcare, Andover, MA, USA) with multiple projections for each coronary artery.

Two experienced interventional cardiologists independently reviewed coronary angiograms and were blinded to clinical and echocardiographic data. We quantified angiographic CAD burden using the Duke CAD Index, a validated prognostic scoring system that integrates the extent and severity of coronary stenoses, including the number of diseased vessels and involvement of prognostically important segments such as the left main and proximal left anterior descending artery.

The Duke CAD Index was calculated according to the validated methodology established by Mark et al., which assigns prognostic scores based on anatomical extent and lesion severity. The scoring system evaluates the total number of major epicardial vessels demonstrating at least 75% stenosis. It incorporates specific weighting for left main coronary artery involvement, which is categorized as two-vessel disease. Furthermore, the calculation accounts for disease involving the proximal left anterior descending artery and differentiates lesion severity by assigning lower scores to stenoses ranging from 50% to 74% compared with those with 75% or greater obstruction.

The Duke CAD Index was chosen for this study because it reflects global atherosclerotic burden and has been widely used for risk stratification and prediction of cardiovascular outcomes. In contrast, the SYNTAX score primarily quantifies anatomical complexity to guide revascularization decisions. We analyzed the index as both a continuous variable and categorized it into tertiles (low, intermediate, and high). Severe CAD was defined as belonging to the highest tertile.

Inter-observer agreement for Duke CAD Index classification was assessed using Cohen's kappa. The kappa coefficient for tertile classification was 0.82 (95% CI 0.71-0.93), indicating excellent agreement. Disagreements were resolved by consensus or discussion with a third reviewer.

Revascularization strategy assessment

We classified revascularization strategies as PCI, CABG, or conservative management. The revascularization decision was made by the institutional heart team based on angiographic findings, clinical presentation, comorbidities, and current guideline recommendations.

Statistical analysis

Based on the observed variability (pooled SD approximately 18), the available sample size (n = 98) was expected to provide approximately 80% power to detect a between-group difference of approximately 12.5 Duke CAD Index points at a two-sided alpha of 0.05.

We present continuous variables as mean ± SD and categorical variables as counts and percentages. We performed group comparisons using independent t-tests or χ² tests as appropriate. We assessed correlations using Spearman's rank correlation coefficient.

We performed multivariable linear regression to identify independent predictors of Duke CAD Index values. We selected variables for the model based on clinical relevance and univariate associations (p < 0.10). Due to the limited sample size and lack of systematic recording, variables such as body mass index, creatinine, uric acid, and medication use could not be incorporated. We performed ordinal logistic regression (proportional odds model) for Duke CAD Index categories. We evaluated the proportional odds assumption using the test of parallel lines.

Model diagnostics were performed to ensure statistical rigor and model validity across all regression analyses. For multivariable linear regression models, residual normality was evaluated using the Shapiro-Wilk test, homoscedasticity was assessed via the Breusch-Pagan test, and multicollinearity was checked by calculating variance inflation factors. For ordinal logistic regression, the proportional odds assumption was evaluated and verified using the test of parallel lines. To further test the robustness of our findings, three distinct sensitivity analyses were conducted. First, we compared patients with HHD directly to hypertensive patients without LVH (n = 30), thereby excluding normotensive individuals from the comparator group. Second, we performed an analysis completely excluding patients with diabetes mellitus (n = 17). Third, we conducted a stratified analysis by smoking status to evaluate whether the observed associations remained consistent across subgroups.

We constructed a receiver operating characteristic (ROC) curve to assess the discriminative ability of the multivariable model (including age, sex, diabetes mellitus, and HHD) for severe CAD. We calculated the area under the curve (AUC) with a 95% CI and determined the model's classification performance (accuracy, sensitivity, and specificity) at a cut-off probability of 0.50.

Given the exploratory nature of this study, we did not adjust for multiple comparisons. However, readers should be aware that the p-values presented are nominal and may be subject to Type I error. Data completeness was high for the primary variables (100%). Dyslipidemia data were available for 97 patients (one missing in the non-HHD group). Other variables (body mass index, creatinine, uric acid, medication use) were not systematically recorded and could not be included.

We considered a p-value <0.05 statistically significant. We conducted analyses using SPSS Statistics version 29.0 (IBM Corp. Released 2022. IBM SPSS Statistics for Windows, Version 29.0. Armonk, NY). The SPSS output files for all primary and sensitivity analyses are available from the corresponding author upon reasonable request.

Ethical considerations

The Institutional Ethics Committee of Cantonal Hospital Zenica approved the study protocol (approval number: 00-03-35-27-11/26, dated January 30, 2026). We anonymized all data before analysis and conducted the study in accordance with the Declaration of Helsinki (2013 revision). Due to the retrospective and anonymized nature of the study, the ethics committee waived the requirement for written informed consent.

## Results

Baseline characteristics

Baseline demographic and clinical characteristics are shown in Table 1. Patients with and without HHD were comparable in age and lipid parameters. Diabetes mellitus was numerically more prevalent among patients with HHD, although this difference did not reach statistical significance. Importantly, diabetes mellitus duration and insulin therapy use did not differ significantly between groups, suggesting that the observed associations were not driven by differences in diabetes mellitus severity.

**Table 1:** Baseline characteristics of patients with and without HHD *Data are presented for patients with diabetes mellitus only (non-HHD: n = 6; HHD: n = 11). Data are presented as mean ± SD or n (%). Comparisons: continuous variables by independent t-test, categorical variables by χ² test. Dyslipidemia data were available for 97 patients; prevalence was similar between groups (p = 0.86). LDL: low-density lipoprotein; HDL: high-density lipoprotein, SD: standard deviation

Variable	Non-HHD (n = 53)	HHD (n = 45)	Test statistic	p-value
Age, years (mean ± SD)	59.4 ± 9.2	60.4 ± 8.1	t = 0.59	0.55
Male sex, n (%)	15 (28.3)	7 (15.6)	χ² = 2.20	0.14
Current smoking, n (%)	43 (81.1)	19 (42.2)	χ² = 15.2	<0.001
Diabetes mellitus, n (%)	6 (11.3)	11 (24.4)	χ² = 2.85	0.09
Duration of diabetes mellitus, years (mean ± SD)*	7 ± 4 (n=6)	10 ± 6 (n=11)	Mann-Whitney U = 40.5	0.60
Insulin therapy, n (%)*	4 (66.7) (n=6)	3 (27.3) (n=11)	Fisher's exact	0.33
Hypertension, n (%)	30 (56.6)	45 (100)	χ² = 25.53	<0.001
LDL cholesterol, mmol/L (mean ± SD)	3.44 ± 1.06	3.32 ± 0.86	t = 0.56	0.58
Total cholesterol, mmol/L (mean ± SD)	5.55 ± 1.25	5.36 ± 1.00	t = 0.20	0.84
HDL cholesterol, mmol/L (mean ± SD)	1.12 ± 0.24	1.06 ± 0.30	t = 1.10	0.33
LDL/HDL ratio	3.16 ± 1.04	3.35 ± 1.16	t = 0.81	0.42
Total cholesterol/HDL ratio	5.13 ± 1.44	5.40 ± 1.76	t = 0.83	0.41

The predominance of female patients in this cohort (71.7% overall) is unusual for angiography cohorts and may reflect specific local referral patterns at our institution. This may limit generalizability to other populations with different referral patterns.

Current smoking was paradoxically more prevalent among non-HHD patients (81.1% vs. 42.2%, p < 0.001) despite non-HHD patients having less angiographic disease. This unexpected finding is likely explained by confounding, selection bias, or chance due to small sample size and is discussed further below.

Echocardiographic characteristics

Echocardiographic findings are presented in Table 2. Left atrial diameter and LVMI were significantly higher in patients with HHD, while LVEF did not differ significantly between groups. Patients with HHD demonstrated significantly higher RWT, consistent with a predominance of concentric hypertrophy phenotypes.

**Table 2:** Echocardiographic parameters according to HHD status All comparisons were made by an independent t-test. LVEF: left ventricular ejection fraction, LV: left ventricular, RWT: relative wall thickness, HHD: hypertensive heart disease, SD: standard deviation

Parameter	HHD (n = 45)	Non-HHD (n = 53)	Test statistic	p-value
LVEF, % (mean ± SD)	52.2 ± 8.1	55.1 ± 6.9	t = 1.62	0.11
Left atrial diameter, mm (mean ± SD)	42.1 ± 5.6	38.8 ± 5.6	t = 3.02	0.003
LV mass index, g/m² (mean ± SD)	133.2 ± 19.1	95.0 ± 23.9	t = 8.75	<0.001
RWT (mean ± SD)	0.455 ± 0.065	0.429 ± 0.08	t = 4.50	<0.001

LV geometry

LV geometry distribution is shown in Table 3. The distribution of LV geometry differed significantly between the groups (p < 0.001), with concentric hypertrophy being the most common phenotype in HHD patients (73.3% vs. 0% in non-HHD).

**Table 3:** LV geometry according to HHD status P-values are calculated by the chi-square test. By definition, all HHD patients had LVH (elevated LVMI), and all non-HHD patients had normal LVMI. LV: left ventricular, HHD: hypertensive heart disease, LVH: left ventricular hypertrophy, LVMI: left ventricular mass index

LV geometry	HHD (n = 45) n (%)	Non-HHD (n = 53) n (%)	p-value
Normal geometry	0 (0.0)	28 (52.8)	<0.001
Concentric remodeling	0 (0.0)	25 (47.2)	<0.001
Eccentric hypertrophy	12 (26.7)	0 (0.0)	0.002
Concentric hypertrophy	33 (73.3)	0 (0.0)	<0.001

Duke CAD Index across LV geometry

Although Duke CAD Index values showed a numerical increase across progressively concentric geometric patterns, these differences did not reach statistical significance (Kruskal-Wallis p = 0.282) (Table 4).

**Table 4:** Duke CAD Index across LV geometry Kruskal-Wallis p = 0.282. LV: left ventricular, CAD: coronary artery disease

LV geometry	Mean rank Duke CAD Index
Normal geometry	41.5
Concentric remodeling	49.6
Eccentric hypertrophy	53.7
Concentric hypertrophy	54.8

Angiographic severity and Duke CAD Index

Patients with HHD exhibited significantly greater angiographic CAD burden, with higher mean Duke CAD Index values compared to patients without HHD (55.8 ± 16.7 vs. 45.3 ± 18.2; p = 0.008) (Table 5).

**Table 5:** Duke CAD Index among patients with and without HHD An independent t-test was used; the data are normally distributed (as confirmed by the Shapiro-Wilk test). CAD: coronary artery disease, HHD: hypertensive heart disease

Variable	Non-HHD (n = 53)	HHD (n = 45)	Test statistic	p-value
Duke CAD Index (mean ± SD)	45.3 ± 18.2	55.8 ± 16.7	t = 2.95	0.008

Vessel-based CAD severity

Vessel-based CAD severity differed significantly between groups (p < 0.001). Patients with HHD had a markedly higher prevalence of multivessel and left main disease, whereas single-vessel disease predominated in patients without HHD (Table 6).

**Table 6:** Vessel-based CAD severity among patients with and without HHD Values are presented as n (%). Comparisons were performed using the Pearson chi-square test. Categories are mutually exclusive. Left main disease was reported separately according to Duke CAD Index methodology; patients with left main involvement were classified according to the Duke CAD Index scoring system, and left main disease is not double-counted with vessel-based categories. CAD: coronary artery disease, HHD: hypertensive heart disease

Variable	Non-HHD (n = 53)	HHD (n = 45)	p-value
1-vessel disease, n (%)	26 (49.1)	4 (8.9)	
2-vessel disease, n (%)	6 (11.3)	19 (42.2)	
3-vessel disease, n (%)	21 (39.6)	18 (40.0)	
Left main disease, n (%)	0 (0.0)	4 (8.9)	<0.001

HHD as an independent predictor

In multivariable linear regression adjusted for age, sex, diabetes mellitus, smoking, and dyslipidemia, HHD remained independently associated with higher Duke CAD Index values (B = 10.33, 95% CI 0.60-20.06; p = 0.038). Age was also independently associated with higher angiographic burden (B = 0.71 per year; p = 0.010). Diabetes mellitus did not reach statistical significance in this model, likely due to limited sample size and residual confounding (Table 7). The lower confidence bound for HHD was close to zero, indicating substantial uncertainty regarding the magnitude of the association.

**Table 7:** Multivariable linear regression analysis for Duke CAD Index Model statistics: R² = 0.175; adjusted R² = 0.120; F = 3.18; p = 0.007. Model diagnostics: Residuals were normally distributed (Shapiro-Wilk p = 0.23) and homoscedastic (Breusch-Pagan p = 0.18). Variance inflation factors were <2.0 for all predictors, indicating no significant multicollinearity. CAD: coronary artery disease, HHD: hypertensive heart disease, CI: confidence interval

Variable	Unstandardized B (SE)	Standardized β	95% CI for B	p-value
HHD	10.33 (4.90)	0.213	0.60-20.06	0.038
Age (per year)	0.71 (0.27)	0.263	0.18-1.25	0.010
Diabetes mellitus	-11.47 (6.26)	-0.185	-23.90-0.96	0.070
Smoking	7.17 (5.03)	0.145	-2.81-17.15	0.157
Dyslipidemia	-0.39 (13.44)	-0.003	-27.03-26.25	0.977
Sex	-8.66 (5.60)	-0.154	-19.74-2.43	0.126
Constant	32.22 (23.09)	-	-13.52-77.96	0.166

The unexpected negative coefficient for diabetes mellitus (B = -11.47) is biologically implausible and almost certainly reflects the small number of diabetic patients (n = 17) and residual confounding rather than a true protective effect. This finding should be interpreted with caution and is not considered clinically meaningful. In a sensitivity analysis excluding diabetic patients, the association between HHD and the Duke CAD Index remained significant (B = 9.73, 95% CI 0.45-19.01; p = 0.041).

Ordinal logistic regression demonstrated that HHD was independently associated with higher odds of belonging to a more severe Duke CAD Index category (OR = 3.19, 95% CI 1.6-9.1; p = 0.015). The proportional odds assumption was not violated (test of parallel lines p = 0.066), although the result approached the conventional significance threshold and should therefore be interpreted cautiously (Table 8).

**Table 8:** Ordinal logistic regression analysis for Duke CAD Index categories Model statistics: Nagelkerke R² = 0.240; test of parallel lines p = 0.066. Duke CAD Index tertiles were defined as follows: low (≤48), intermediate (49-56), and high (≥57). HHD: hypertensive heart disease, CAD: coronary artery disease, OR: odds ratio, CI: confidence interval

Variable	OR	95% CI	p-value
HHD	3.19	1.6-9.1	0.015
Age (per year)	1.08	1.01-1.11	0.001
Male sex	2.15	0.81-5.72	0.126
Diabetes mellitus	0.47	0.14-1.53	0.217

ROC analysis and classification performance for severe CAD

We performed an ROC analysis to evaluate the discriminative ability of a multivariable model (including age, sex, diabetes mellitus, and HHD) for predicting severe CAD (defined as the highest tertile of the Duke CAD Index). The AUC was 0.753 (95% CI 0.655-0.851, p < 0.001). At a cut-off probability of 0.50, the model showed an overall accuracy of 70.7%, sensitivity of 70.2%, and specificity of 71.2% (Table 9).

**Table 9:** ROC analysis for severe CAD prediction Cutoff probability = 0.50. ROC: receiver operating characteristic, CAD: coronary artery disease, AUC: area under the curve, CI: confidence interval

Model	AUC	95% CI	p-value	Accuracy	Sensitivity	Specificity
Without HHD	0.695	0.591-0.798	0.001	63.6%	59.6%	67.3%
With HHD	0.753	0.655-0.851	<0.001	70.7%	70.2%	71.2%

The model including HHD demonstrated numerically higher discriminative ability compared to the model without HHD (AUC 0.753 vs. 0.695). Formal testing of the difference between AUCs (DeLong test) was not performed due to the exploratory nature of the analysis.

**Figure 1:**
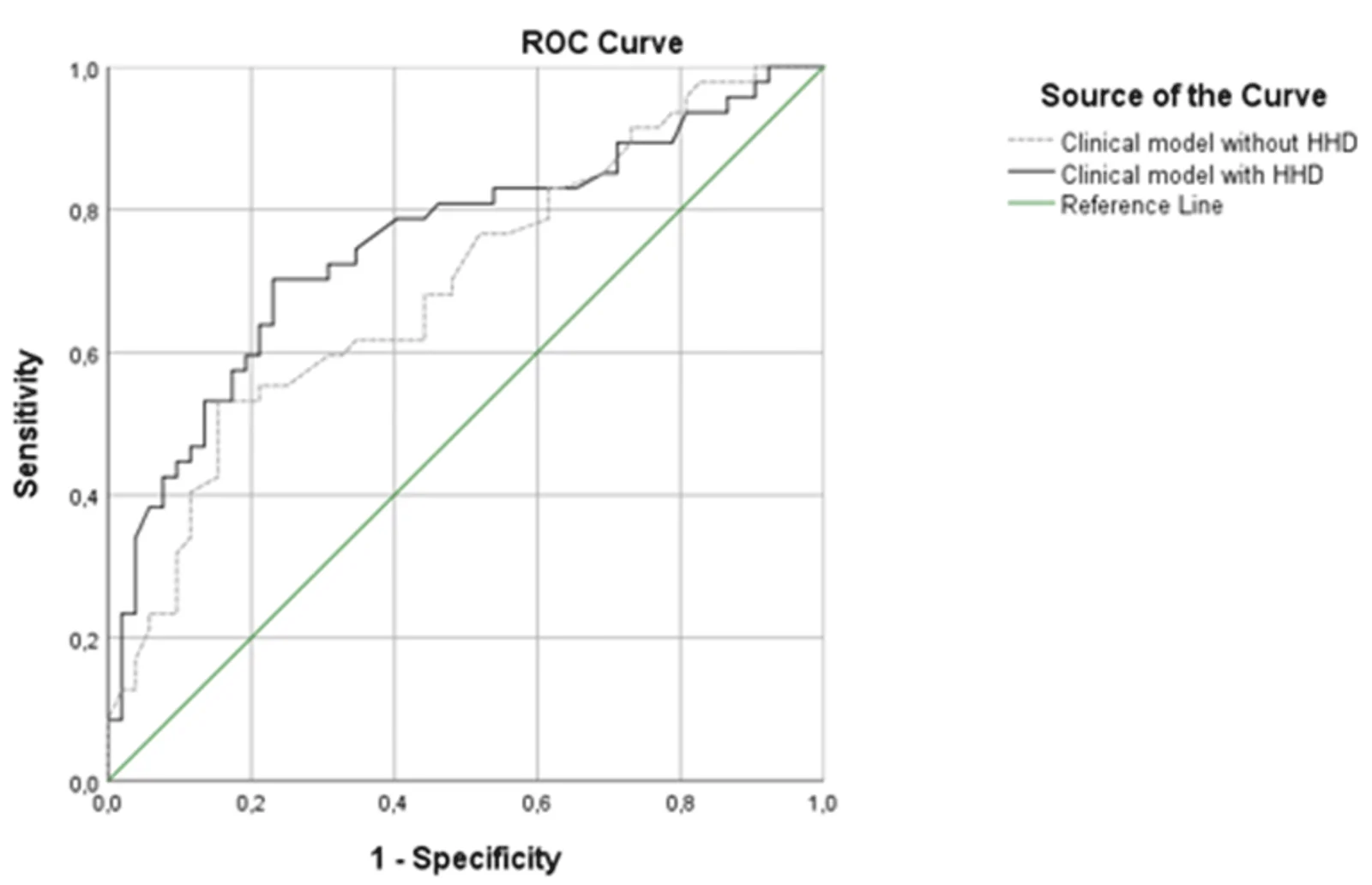
ROC curve for the prediction of severe CAD ROC curve for the multivariable model including HHD, age, sex, and diabetes mellitus (solid line) compared to the model without HHD (dashed line). The model with HHD demonstrated numerically greater discriminative ability (AUC = 0.753, 95% CI 0.655-0.851) than the model without HHD (AUC = 0.695, 95% CI 0.591-0.798). The diagonal line represents the reference (AUC = 0.5). ROC: receiver operating characteristic, CAD: coronary artery disease, HHD: hypertensive heart disease, AUC: area under the curve, CI: confidence interval

Figure 1 shows the ROC curve for the multivariable model including HHD, age, sex, and diabetes mellitus (solid line) compared to the model without HHD (dashed line). The model with HHD demonstrated numerically higher discriminative ability (AUC = 0.753 vs. 0.695).

Correlation analyses

We found no significant correlation between LV mass index and Duke CAD Index (ρ = 0.072, p = 0.479) (Table 10).

**Table 10:** Correlation analysis between angiographic, echocardiographic, and clinical parameters All correlations were assessed using Spearman's rank correlation coefficient. LV: left ventricular, CAD: coronary artery disease, RWT: relative wall thickness, LVEF: left ventricular ejection fraction

Variables	Spearman ρ	p-value
Duke CAD Index category ↔ vessel-based CAD severity	0.552	<0.001
Duke CAD Index ↔ LV mass index	0.072	0.479
Left atrial diameter ↔ LVEF	-0.346	<0.001
Duke CAD Index ↔ RWT	0.076	0.452

Sensitivity analyses

Sensitivity Analysis 1

When patients with HHD were compared only with hypertensive patients without LVH (n = 30), HHD remained significantly associated with a higher Duke CAD Index (B = 9.87, 95% CI: 0.12-19.62; p = 0.047). This finding confirms that the observed association is not merely attributable to the inclusion of normotensive patients in the comparator group.

Sensitivity Analysis 2

After excluding all patients with diabetes mellitus (n = 17), the association between HHD and the Duke CAD Index remained significant (B = 9.73, 95% CI: 0.45-19.01; p = 0.041), suggesting that the observed relationship was not driven by the presence of diabetes mellitus.

Sensitivity Analysis 3

Stratification by smoking status demonstrated that the association between HHD and the Duke CAD Index remained significant in both smokers (B = 8.96, 95% CI: 0.21-17.71; p = 0.045) and non-smokers (B = 11.23, 95% CI: 1.34-21.12; p = 0.027). These findings indicate that smoking status did not confound the primary association between HHD and CAD severity.

Revascularization strategy

Direct Analysis by HHD Status

Revascularization strategy did not differ significantly between HHD and non-HHD groups (Table 11).

**Table 11:** Revascularization strategy according to HHD status PCI: percutaneous coronary intervention, CABG: coronary artery bypass grafting, HHD: hypertensive heart disease

Revascularization strategy	Non-HHD (n = 53)	HHD (n = 45)	Test statistic	p-value
PCI, n (%)	38 (71.7)	32 (71.1)	χ² = 0.004	0.95
CABG, n (%)	9 (17.0)	9 (20.0)	χ² = 0.15	0.70
Conservative, n (%)	6 (11.3)	4 (8.9)	χ² = 0.16	0.69

Analysis by Duke CAD Index Categories

Distribution of revascularization strategies across Duke CAD Index categories is shown in Table 12. Although CABG was numerically more frequent in higher Duke CAD Index categories, this association did not reach statistical significance (p = 0.299). The lack of a significant association may reflect the limited sample size and the fact that treatment decisions were individualized by the heart team, considering not only angiographic severity but also clinical presentation, comorbidities, and guideline-based indications. In this cohort, PCI was the predominant strategy even in high-risk categories, consistent with contemporary real-world trends favoring PCI in many patients with multivessel disease.

**Table 12:** Revascularization strategy according to Duke CAD Index category Pearson χ² test: χ² = 4.887, df = 4, p = 0.299. PCI: percutaneous coronary intervention, CABG: coronary artery bypass grafting, CAD: coronary artery disease

Duke CAD Index category	PCI n (%)	CABG n (%)	Conservative n (%)	Total
Category 1 (low)	20 (71.4)	4 (14.3)	4 (14.3)	28
Category 2 (intermediate)	18 (75.0)	2 (8.3)	4 (16.7)	24
Category 3 (high)	32 (69.6)	12 (26.1)	2 (4.3)	46
Total	70 (71.4)	18 (18.4)	10 (10.2)	98

## Discussion

Summary of findings

In this hypothesis-generating observational study, we found that HHD, defined as hypertension with echocardiographic LVH, was significantly associated with greater angiographic CAD burden as quantified by the Duke CAD Index. Patients with HHD had higher mean Duke CAD Index scores compared with those without HHD, and HHD was independently associated with severe CAD, with more than threefold higher odds. These findings remained significant after adjustment for traditional risk factors and in multiple sensitivity analyses, underscoring the clinical relevance of HHD as a marker of advanced coronary pathology. Notably, diabetes mellitus duration and insulin therapy did not differ between groups, suggesting that the observed association is unlikely to be explained by diabetes mellitus severity alone. Nevertheless, the overlap between hypertensive and diabetic cardiomyopathy cannot be fully disentangled in this retrospective study, and prospective investigation is warranted.

The Duke CAD Index was chosen for this study because it provides a validated global assessment of coronary atherosclerotic burden and long-term prognostic risk, as opposed to purely anatomical complexity scores such as the SYNTAX score [15,16]. While the SYNTAX score is specifically designed to guide revascularization strategy by quantifying lesion complexity, the Duke CAD Index captures overall disease extent. It reflects the cumulative impact of epicardial stenoses on prognosis. Recent evidence further supports the Duke CAD Index as a marker of biologically active coronary disease, as it independently correlates with pericoronary adipose tissue attenuation, a CT-derived measure of vascular inflammation [16]. This biological dimension may partly explain why HHD, a systemic hypertensive phenotype, is linked to higher Duke CAD Index scores.

While these findings suggest a clinically meaningful association between HHD and angiographic CAD burden, the study's limited sample size (n = 98) and retrospective design necessitate cautious interpretation. The wide CIs observed in regression analyses indicate unstable estimates that require confirmation in larger, prospectively designed cohorts. The lower confidence bound for HHD in the multivariable model was close to zero, indicating substantial uncertainty regarding the magnitude of the association. As such, the present findings should be considered hypothesis-generating and form the basis for future investigations rather than providing definitive evidence for changes in clinical practice.

HHD as a systemic vascular phenotype

HHD represents more than isolated LVH; it reflects a systemic vascular phenotype encompassing endothelial dysfunction, microvascular rarefaction, and diffuse interstitial fibrosis [3]. Microvascular dysfunction in hypertension is characterized by impaired endothelium-dependent vasodilation, reduced coronary flow reserve, and increased minimal coronary vascular resistance [5]. These microvascular abnormalities are thought to predispose to myocardial ischemia independent of epicardial stenosis severity. Furthermore, the systemic nature of hypertensive vascular disease is supported by the association between HHD and increased pericoronary adipose tissue attenuation, a marker of vascular inflammation [16]. This inflammatory phenotype may partly explain why patients with HHD appear to have more extensive CAD despite comparable levels of traditional risk factors.

Beyond myocardial mass: the phenotypic nature of HHD

A key observation of this study is the lack of a significant correlation between LVMI and the severity of CAD, despite the strong association between HHD as a clinical entity and angiographic disease burden. This paradox emphasizes that HHD is not merely an increase in myocardial mass but a complex pathological phenotype [3,11,14]. HHD encompasses a constellation of structural and functional alterations, including increased myocardial stiffness, reduced compliance, microvascular rarefaction, endothelial dysfunction, and diffuse interstitial fibrosis [3]. Among these, myocardial fibrosis appears to play a central role by impairing coronary flow reserve and increasing ischemic vulnerability, even in the absence of critical epicardial stenoses. Consequently, HHD may serve as a surrogate marker of systemic and coronary microvascular remodeling processes that may be associated with a higher angiographic CAD burden [3,11]. The dissociation between LVMI and Duke CAD Index in our cohort supports the notion that cardiovascular risk is driven more by pathological remodeling than by myocardial mass per se.

Recent data from Halatiu et al., demonstrating the association of the modified Duke CAD Index with the pericoronary fat attenuation index, provide mechanistic support for our findings [16]. This suggests that HHD identifies a vascular inflammatory phenotype characterized by more extensive and biologically active coronary disease, rather than simply greater myocardial mass. These concordant observations reinforce the value of the Duke CAD Index as a contemporary risk stratification tool that integrates anatomical disease extent with underlying biological activity.

Mechanisms linking hypertensive remodeling to atherosclerotic burden

Several interconnected mechanisms are thought to contribute to the observed association between HHD and greater angiographic CAD burden. First, the increased myocardial oxygen demand in HHD, driven by elevated wall stress and mass, may exacerbate ischemia in the presence of stenoses and promote collateral vessel development [14]. Second, reduced coronary flow reserve and impaired vasodilatory capacity may increase ischemic vulnerability even without critical stenoses [7]. Third, hypertension-induced oxidative stress and inflammation may promote endothelial dysfunction and accelerate atherosclerosis [5]. Fourth, myocardial fibrosis increases tissue stiffness, impairs diastolic function, and may promote a pro-inflammatory milieu that accelerates vascular disease [15]. These mechanisms likely operate synergistically, explaining why HHD patients have greater disease burden independent of LV mass alone.

Importance of LV geometry

Our findings are further supported by literature emphasizing LV geometry as a superior predictor of cardiovascular risk compared with absolute myocardial mass [13]. Concentric remodeling characterized by increased RWT with preserved or modestly increased LV mass has been consistently associated with adverse outcomes, including stroke and coronary events. Concentric hypertrophy, in which both LV mass and RWT are increased, represents the most malignant phenotype [13].

The association between HHD and severe CAD observed in this study is concordant with data indicating that concentric phenotypes are linked to impaired coronary microcirculation and heightened ischemic risk. A correlation between patients with HHD and microvascular dysfunction probably exists, potentially predisposing them to more extensive and diffuse CAD, as shown in a study by Halatiu et al. [12,15,17].

Of note, the absence of an independent association between RWT or LV geometry and angiographic disease burden should not be interpreted as a lack of biological relevance. Rather, these parameters may represent intermediate phenotypic expressions of a broader hypertensive disease process.

Aging, remodeling, and the LV mass paradox

Although age was confirmed as an independent predictor of CAD severity in our multivariable model, population-based studies have paradoxically shown that LV mass often decreases with advancing age. This apparent contradiction can be reconciled by examining age-related changes in ventricular geometry. Data from large cohorts such as MESA and studies by Marwick and colleagues demonstrate that both LV mass and volumes decline with age; nonetheless, ventricular volumes decrease more rapidly than mass [14]. This disproportionate reduction results in an increased mass-to-volume ratio and elevated RWT, favoring concentric remodeling rather than overt hypertrophy.

Therefore, in older individuals, the burden of coronary disease is not necessarily linked to increased myocardial mass but rather to maladaptive geometric remodeling, myocardial fibrosis, and cumulative vascular injury. The lack of correlation between LVMI and Duke CAD Index in our study reinforces this concept, suggesting that severe CAD can coexist with stable or even declining LV mass, particularly in elderly patients. In this context, geometry and tissue composition, rather than mass alone, become dominant determinants of cardiovascular risk.

Revascularization strategies in patients with HHD

The treatment distribution in our cohort, with PCI predominating (71.4%) even among patients with the highest Duke CAD Index, mirrors contemporary practice patterns reported in large registries, where PCI accounts for the majority of revascularization procedures, partly due to advances in drug-eluting stents [17,18]. The lack of significant differences in treatment strategy by either HHD status or Duke CAD Index category likely reflects the complex, individualized nature of heart team decisions, which incorporate not only angiographic severity but also clinical presentation, comorbidities, patient preferences, and guideline-based indications. Furthermore, while validated for risk stratification, the Duke CAD Index is not specifically designed to guide revascularization strategy selection, a role for which the SYNTAX score is better suited [18-20]. The absence of a significant association should not be interpreted as evidence that angiographic severity is irrelevant to treatment decisions, but rather that in this small cohort, multiple factors influenced the heart team's recommendations.

Smoking paradox

The smoking paradox observed in this cohort, where smoking was more prevalent in the group with less CAD, is unexpected and deserves comment. Several explanations are plausible. First, this may reflect residual confounding, as smoking was more prevalent in non-HHD patients who also had lower blood pressure and no LVH. Second, the cross-sectional design may introduce survival bias, where smokers with HHD may have been less likely to be included due to higher mortality. Third, the small sample size increases the likelihood of chance associations. This paradoxical finding underscores the limitations of observational studies with small samples and the importance of comprehensive adjustment for potential confounders.

Discriminative ability of the multivariable model

Our ROC analysis showed that a model including HHD, age, sex, and diabetes mellitus had moderate discriminative ability for severe CAD (AUC = 0.753). The model including HHD demonstrated numerically higher discriminative ability compared to the model without HHD (AUC 0.753 vs. 0.695). This suggests that HHD, in combination with other clinical variables, may help identify patients at higher risk of extensive coronary disease. The model's classification performance (accuracy 70.7%, sensitivity 70.2%, specificity 71.2%) indicates potential for further investigation. Despite this, validation in larger cohorts is needed, ideally with the inclusion of additional risk markers such as renal function, obesity indices, and cardiac biomarkers.

Clinical implications

Our findings carry an important clinical message. While traditional risk factors remain the cornerstone of primary prevention, our data suggest that in patients undergoing evaluation for suspected CAD, the presence of echocardiographic evidence of HHD, irrespective of the conventional risk factor burden, identifies a subgroup with disproportionately severe and diffuse coronary disease. This implies that the absence of multiple traditional risk factors should not be interpreted as low risk if HHD is present. Rather, HHD appears to serve as an integrative biomarker of cumulative cardiovascular injury, reflecting not only the duration and severity of hypertension but also the integrated effects of hemodynamic stress, neurohormonal activation, and microvascular dysfunction on the myocardium. Therefore, we propose that echocardiographic detection of HHD in clinical practice should prompt heightened clinical vigilance for significant underlying CAD, even in patients who do not present with an overtly unfavorable traditional risk factor profile. These findings are exploratory and require prospective validation before incorporation into clinical algorithms.

Limitations

This study has several important limitations that should be considered when interpreting the findings.

Study Design and Power

The retrospective, single-center design with a relatively small sample size (n = 98) limits generalizability and precludes causal inference. The study is substantially underpowered, as reflected in the wide CIs for several estimates, and should be considered hypothesis-generating. A post hoc power calculation showed that the study had adequate power for the primary comparison (80% to detect a 12.5-point difference in the Duke CAD Index), but not for subgroup analyses or complex multivariable modeling. With only 45 patients in the HHD group and 33 patients in the highest Duke CAD Index tertile, the events-per-variable ratio is marginal, and estimates are unstable. This is particularly evident for diabetes mellitus, which showed an implausible protective direction with wide CIs, almost certainly reflecting small numbers (17 diabetics) and residual confounding rather than a true biological effect.

Confounding and Missing Variables

Several potentially important variables, including body mass index, serum creatinine, serum uric acid, detailed medication adherence profiles, diastolic function parameters, left atrial volume index, and natriuretic peptide levels, were not systematically recorded and could not be incorporated into multivariable models. Residual confounding, especially from the interplay between diabetes mellitus and hypertension in causing LVH (diabetic cardiomyopathy), cannot be fully excluded. The unexpected direction of association for diabetes mellitus in our models highlights the instability of estimates when the number of events is limited.

Measurement Limitations

The Duke CAD Index, while validated for angiographic assessment, does not evaluate plaque composition, total atheroma volume, or microvascular function. Standard echocardiography was used to define LVH, but more refined imaging modalities (global longitudinal strain, cardiac magnetic resonance with extracellular volume quantification) might provide additional prognostic information and help explain the dissociation between LVMI and angiographic severity observed in this study.

LV Geometry Consideration

Because LVH formed part of the HHD definition, analyses involving LV geometry should be considered descriptive. They cannot establish independent prognostic effects of ventricular geometry beyond the HHD phenotype itself. The observed association between HHD and CAD severity should not be interpreted as evidence that ventricular geometry independently predicts CAD burden.

Selection Bias

Patients undergoing coronary angiography represent a selected population with suspected or established stable CAD, which may limit generalizability to the general hypertensive population. The unusual sex distribution (predominantly female) may reflect local referral patterns and should be considered when generalizing findings to other populations.

Analytical Limitations

The model events-per-variable ratio is borderline, and the unexpected direction of association for diabetes mellitus suggests unstable estimates due to small numbers. The lack of adjustment for multiple comparisons means some findings may be due to chance. The smoking paradox observed in this cohort may reflect residual confounding, selection bias, or chance due to small sample size.

Several clinical parameters relevant to hypertension, such as the exact duration of the disease, the adequacy of long-term blood pressure control, and specific antihypertensive drug classes, were not available for analysis. This limitation is inherent to the retrospective design of our study, as the parent database was primarily designed to evaluate CAD severity. Consequently, our findings should be interpreted with these missing covariates in mind, and future prospective studies are needed to address these specific determinants.

Temporal Limitations

The cross-sectional design precludes temporal assessment of the relationship between HHD development and CAD progression. We cannot determine whether HHD preceded CAD or whether both developed concurrently, nor can we assess the trajectory of CAD severity over time in relation to HHD.

Generalizability

The predominance of female patients (71.7%) and the specific referral patterns at our single center may limit generalizability to other populations with different demographic characteristics and clinical practices.

Given these limitations, the present study should be viewed as hypothesis-generating, and the observed associations require confirmation in larger prospective cohorts before any clinical recommendations can be made.

## Conclusions

Although HHD is a well-established clinical entity, systematic quantification of angiographic CAD burden specifically in this population, particularly using the Duke CAD Index, remains remarkably scarce in the current literature. In this hypothesis-generating observational study, HHD was associated with a high-risk phenotype characterized by greater angiographic CAD burden and adverse cardiac remodeling, irrespective of the traditional cardiovascular risk factor profile. Critically, our findings highlight that the absence of multiple traditional risk factors (e.g., diabetes mellitus, dyslipidemia, smoking, or poorly controlled hypertension) should not provide false reassurance when echocardiographic signs of HHD are present, as HHD appears to signal advanced cumulative cardiovascular injury. A clinical model incorporating HHD, age, sex, and diabetes mellitus demonstrated moderate discriminative ability for severe CAD. In this limited cohort, angiographic disease severity did not significantly influence revascularization strategy selection, likely reflecting individualized clinical decision-making and the use of an index primarily designed for risk stratification rather than anatomical complexity. These observational findings should be interpreted with caution and require confirmation in larger prospective studies with comprehensive risk factor, biomarker, and advanced imaging phenotyping. Pending such validation, the potential clinical utility of HHD as a risk-stratification tool warrants further evaluation; nevertheless, our study provides an important hypothesis-generating framework that underscores HHD as a potentially underestimated marker of severe coronary atherosclerosis.
